# Sulforaphane inhibits angiotensin II-induced cardiomyocyte apoptosis by acetylation modification of Nrf2

**DOI:** 10.18632/aging.204247

**Published:** 2022-08-23

**Authors:** Huanhuan Wang, Ge Yang, Yuan Tian, Jinjie Li, Lingbin Meng, Xin Jiang, Ying Xin

**Affiliations:** 1 Jilin Provincial Key Laboratory of Radiation Oncology and Therapy, The First Hospital of Jilin University, Changchun 130021, Jilin, China; 2 Department of Radiation Oncology, The First Hospital of Jilin University, Changchun 130021, Jilin, China; 3 NHC Key Laboratory of Radiobiology, School of Public Health, Jilin University, Changchun 130021, Jilin, China; 4 Key Laboratory of Pathobiology, Ministry of Education, Jilin University, Changchun 130021, Jilin, China; 5 Department of Hematology and Medical Oncology, Moffitt Cancer Center, Tampa, FL 33612, USA

**Keywords:** sulforaphane, nuclear factor erythroid 2-related factor, oxidative stress, angiotensin II

## Abstract

Oxidative stress is the central cause of angiotensin II (Ang II)-induced myocardial injury, and nuclear factor erythroid 2-related factor (Nrf2) is the core molecule of the anti-oxidant defense system. We have previously demonstrated that sulforaphane (SFN) can prevent Ang II-induced myocardial injury by activating Nrf2; however, the underlying molecular mechanism is still unclear. This study aimed to evaluate whether SFN prevents Ang II-induced cardiomyocyte apoptosis through acetylation modification of *Nrf2*. Wild-type and *Nrf2* knockdown embryonic rat cardiomyocytes (H9C2) were exposed to Ang II to induce apoptosis, oxidative stress, and inflammatory responses. SFN treatment significantly reduced Ang II-induced cardiomyocyte apoptosis, inflammation and oxidative stress. Activation of Nrf2 played a critical role in preventing cardiomyocyte apoptosis. After Nrf2 was knockdown, the anti-inflammatory, antioxidant stress of SFN were eliminated. Furthermore, Nrf2 activation by SFN was closely related to the decreased activity of histone deacetylases (HDACs) and increased histone-3 (H3) acetylation levels in *Nrf2* promoter region. These findings confirm that Nrf2 plays a key role in SFN preventing Ang II-induced cardiomyocyte apoptosis. SFN activates Nrf2 by inhibiting HDACs expression and activation.

## INTRODUCTION

Cardiovascular diseases are the main cause of death worldwide, accounting for 32% of the total number [1]. The renin-angiotensin-aldosterone system (RAAS) plays a central role in the occurrence of many cardiovascular diseases by compromising vascular tension, electrolyte balance, and sympathetic nervous activity [2, 3]. Angiotensin II (Ang II), a main effector molecule of the RAAS, plays a crucial role in the occurrence of various cardiomyopathy, such as diabetic cardiomyopathy [4], alcoholic cardiomyopathy [5], and ischemia-reperfusion injury [6]. Ang II binds to its receptor AT1 or AT2 to activate nicotinamide adenine dinucleotide phosphate oxidase (NOX) and produce a large number of reactive oxygen species (ROS) [4], which leads to oxidative stress [5, 7] when the scavenging capacity of the body is exceeded. Excessive ROS inactivate various proteins, leading to cardiomyocyte apoptosis [8, 9] and subsequently loss of contractile tissue and initiation of cardiac remodeling and cardiomyopathy [10, 11]. In addition, excessive ROS could also promote myocardial inflammation and fibrosis to aggravate myocardial apoptosis and cardiac dysfunction by activating epidermal growth factor receptor and NF-κB signaling pathways [12]. Previous studies have revealed that upregulating the expression of intracellular antioxidant enzymes, including thioredoxin 2 (Txn-2) and heme oxygenase-1 (HO-1), could increase the antioxidant capacity of myocardial cells and inhibit myocardial injury and ventricular remodeling [13, 14]. However, clinical trials have revealed that non-selective ROS clearance by ROS scavenger agents is ineffective in the treatment of cardiovascular disease [15, 16], which implies that upregulation of the endogenous antioxidant stress system may be an effective strategy.

Nuclear factor erythroid 2-related factor (Nrf2) is a crucial transcription factor for antioxidant stress *in vivo*. Under physiological conditions, the activity of Nrf2 is mainly regulated by its negative regulator Keap1. Nrf2 binding to the Kelch domain of Keap1 mediates its ubiquitination and proteasome degradation [17]. When the body is under oxidative stress, Nrf2 dissociates from Keap1 and is translocated to the nucleus, where it binds to the antioxidant stress element (ARE) in the promoter region of downstream genes. Hereby, the expression of downstream antioxidant genes, including NAD(P)H oxidoreductase (*NQO1*), *HO1*, and catalase (*CAT*), is upregulated to ameliorate oxidative stress and inhibit cell apoptosis [18, 19]. Nrf2 upregulation can reportedly prevent a variety of oxidative stress-related myocardial injuries. For example, Nrf2 agonists reduce endotoxin-induced myocardial injury [20]. Additionally, activation of Nrf2/ARE effectively prevents diabetic cardiomyopathy [21], myocardial ischemia-reperfusion injury [22, 23], and doxorubicin-induced cardiotoxicity [24]. Moreover, our previous study confirmed that Nrf2 upregulation effectively protects against Ang II-induced myocardial injury [25]. These results suggest that Nrf2 may be an effective target to prevent myocardial oxidative stress and apoptosis by activating the endogenous antioxidant system.

Sulforaphane (SFN) is an isothiocyanate compound extracted from cruciferous vegetables, including cauliflower and broccoli. As an Nrf2 agonist, SFN exhibits antioxidant capacity [26, 27]. Unlike synthetic Nrf2 agonists, SFN is derived from natural vegetables and is safer. Indeed, SFN prevents myocardial ischemia-reperfusion injury [28], diabetic cardiomyopathy and nephropathy [29], and Ang II-induced cardiomyopathy by upregulating Nrf2 [25]. However, the mechanism underlying Nrf2 activation by SFN in cardiomyocytes requires further exploration.

It has been reported that SFN could modify gene expression through epigenetic modification. This is because SFN acts as an inhibitor of histone deacetylases (HDACs) and upregulates the expression of several antitumor genes, such as p21 and Bax [30–32]. In prostate cancer model, SFN promotes Nrf2 expression by inhibiting CpG island methylation of *Nrf2* promoter region [33]. In addition, SFN has also been shown to inhibit histone acetylation in the *Nrf2* promoter region in skin tumor transformation [34]. Therefore, this study intended to confirm the protective effect of upregulated Nrf2 in cardiomyocytes and explore Nrf2 activation by sulforaphane-mediated acetylation modification. This study provides theoretical and experimental basis for the prevention and treatment of Ang II-induced myocardial injury.

## RESULTS

### SFN prevents Ang II-induced cardiomyocyte apoptosis

**Figure 1:**
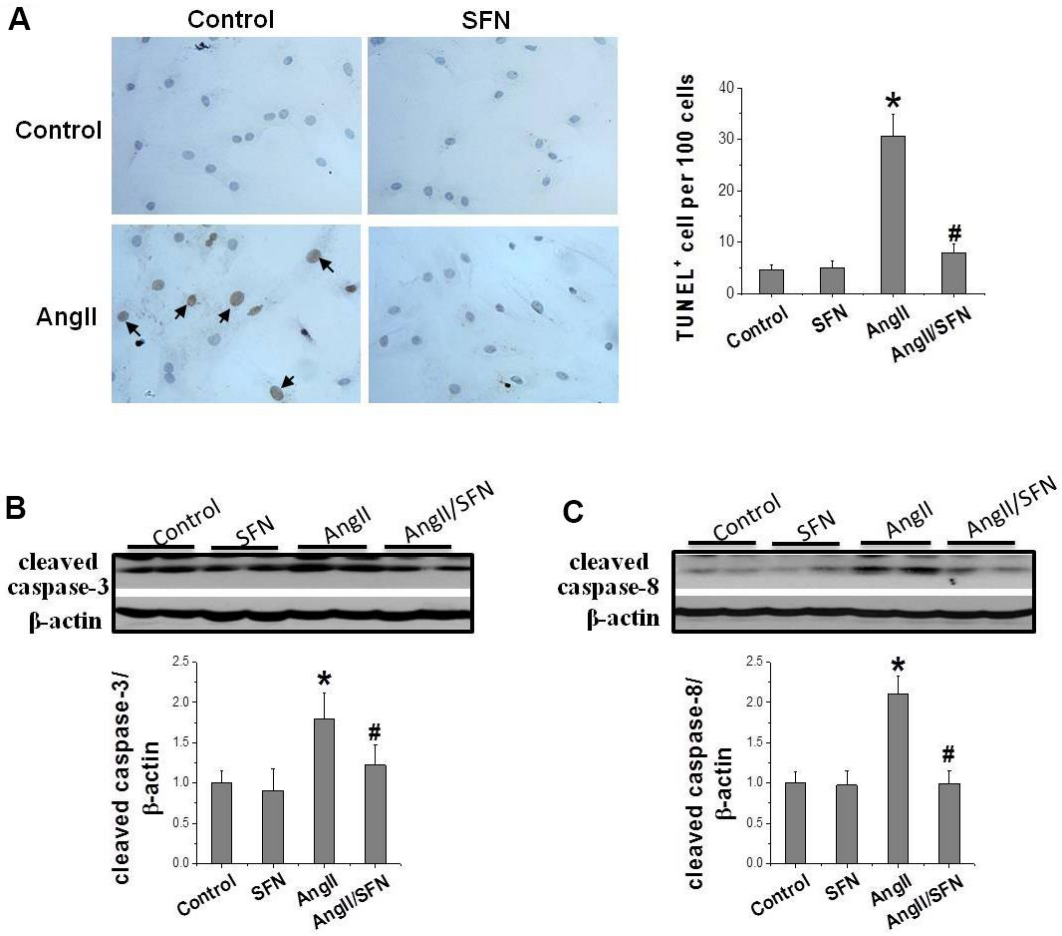
**SFN prevents Ang II-induced cardiomyocytes apoptosis.** TUNEL staining was used to detect the number of apoptotic cells (brownish-yellow particles in the nucleus) (**A**). Western blot was used to detect the expression of cleaved caspase-3 (**B**), cleaved caspase-8 (**C**). Data were presented as the mean SD (n = 3). *P < 0.05 vs control; # *P* < 0.05 vs Ang II.

In order to confirm the preventive effect of SFN on cardiomyocyte apoptosis, TUNEL staining was performed to detect the number of apoptotic cells, and western blotting was applied to detect the expression of apoptotic proteins, respectively. The results revealed that Ang II significantly increases cell apoptosis compared with the control group. Meanwhile, the number of apoptotic cells in the Ang II/SFN group was significantly lower compared with Ang II group (Figure 1A). Consistent with this, Ang II significantly upregulated the expression of apoptosis-related proteins cleaved caspase-3 and cleaved caspase-8 compared with the control group. The expression of cleaved caspase-3 and cleaved caspase-8 were significantly lower in the Ang II/SFN group compared with Ang II group (Figure 1B, 1C). These findings confirm that SFN could protect against Ang II-induced cardiomyocyte apoptosis.

### SFN inhibits Ang II-induced inflammation and oxidative stress

**Figure 2:**
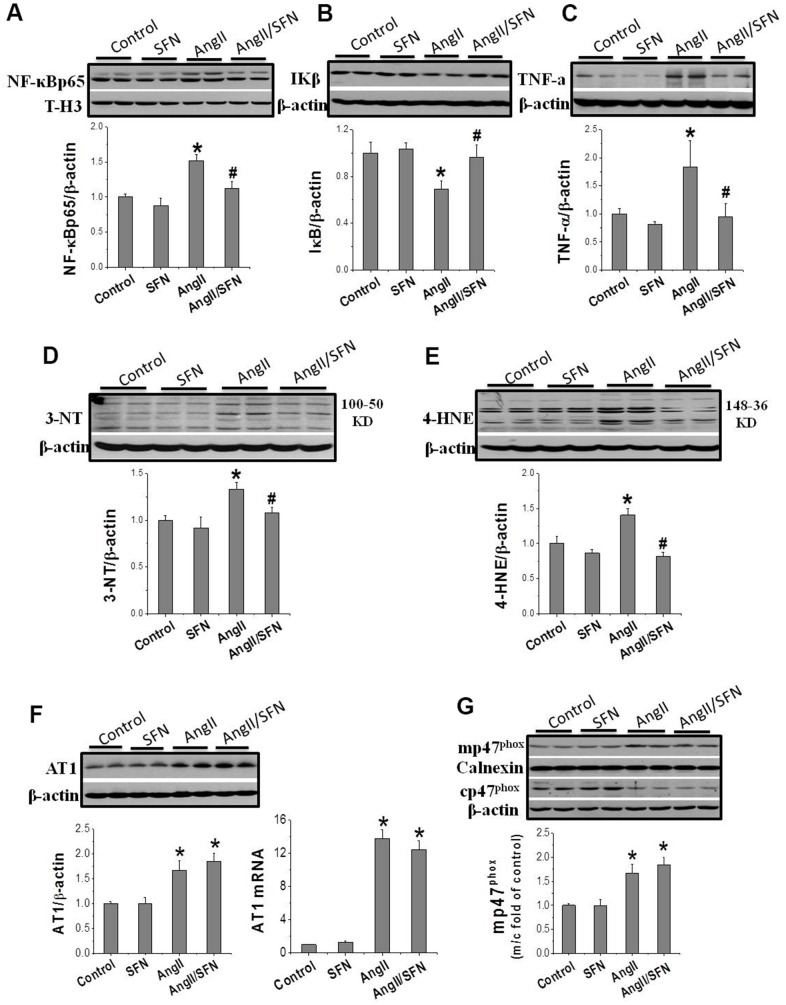
**SFN inhibits Ang II-induced inflammation and oxidative stress.** Western blot was used to detect the expression of inflammatory factors NF-κB (**A**), IKβ (**B**), TNF-α (**C**), oxidative stress indicators 3-NT (**D**), 4-HNE (**E**). Data were presented as the mean SD (n = 3). SFN has no effect on the activation of AT1 and NOX. Western blot and qPCR were used to detect the AT1 expression (**F**). Western blot was used to detect the expression of mp47phox and cp47phox (**G**). Data were presented as the mean SD (n = 3). *P < 0.05 vs control; # *P* < 0.05 vs Ang II.

Inflammation and oxidative stress are the two important factors of Ang II-induced cardiomyocyte apoptosis that aggravate each other. The expression of the inflammatory factors NF-κBp65, IKβ, and tumor necrosis factor (TNF)-α and oxidative stress related indicators 3-nitrotyrosine (3-NT) and 4-hydroxy-2-nonenal (4-HNE) were determined by western blotting. Ang II significantly increased the expression of the pro-inflammatory factors NF-κBp65 and TNF-α and dramatically decreased the expression of the anti-inflammatory factor IKβ compared to the control group. On the contrary, the expression of NF-κBp65 and TNF-α were significantly inhibited and that of IKβ was significantly enhanced in the Ang II/SFN group compared to the Ang II group (Figure 2A–2C). Meanwhile, compared to the control group, the expression of 3-NT and 4-HNE in the Ang II group were significantly upregulated. However, in the Ang II/SFN group, their expression was significantly lower than in the Ang II group (Figure 2D, 2E). Collectively, these findings suggested that SFN may prevent myocardial injury by inhibiting Ang II-induced inflammation and oxidative stress.

### SFN upregulates Nrf2 expression and function

The results confirm that SFN protect against Ang II-induced cardiomyocyte apoptosis and is closely associated with the inhibition of inflammation and oxidative stress. To further explore whether SFN exerts protective effect by inhibiting the AT1 expression and NOX enzymes activation, we performed real-time PCR (qPCR) and western blotting to detect the mRNA and protein expression levels of AT1 and p47^phox^. AT1 expression was significantly upregulated in the Ang II group at the mRNA and protein levels, and was not affected by SFN treatment (Figure 2F). The cytoplasmic subunit cp47^phox^ is phosphorylated and translocated to the cell membrane (mp47^phox^) to form an active NOX complex. Ang II significantly upregulated the ratio of mp47^phox^ to cp47^phox^ expression. However, there was no difference of the ratio between the Ang II and Ang II/SFN groups (Figure 2G). The data suggested that SFN does not play its antioxidative role by inhibiting AT1 and NOX.

**Figure 3:**
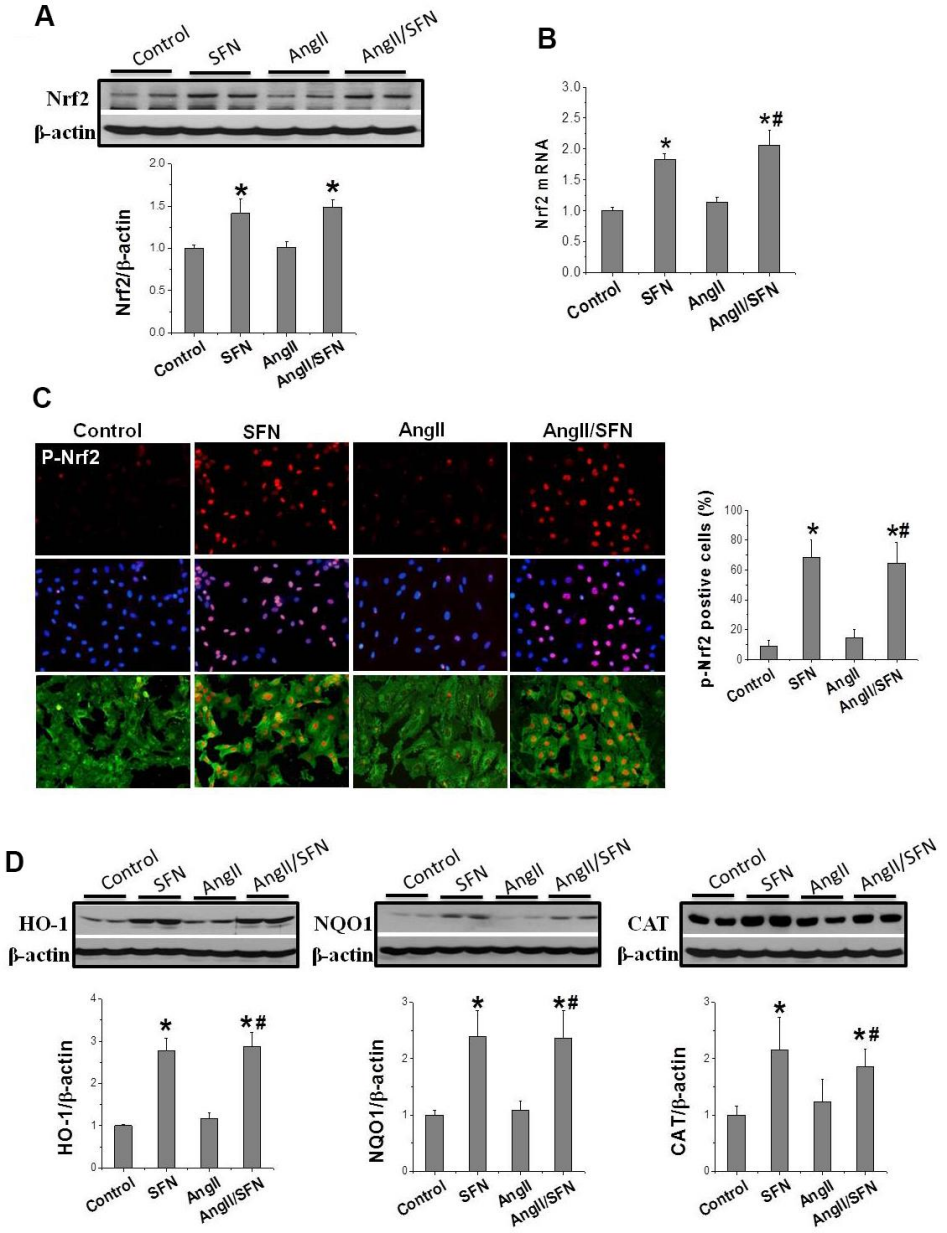
**SFN up-regulates Nrf2 expression and function.** Western blot (**A**), RT-qPCR (**B**) were used to detect Nrf2 protein and mRNA levels. Immunofluorescence staining was applied to detect p-Nrf2 expression and distribution (red) (**C**). Western blot was used to detect the expression of Nrf2 downstream antioxidant genes *HO-1, NQO1, CAT* (**D**). Data were presented as the mean SD (n = 3). *P < 0.05 vs control; # *P* < 0.05 vs Ang II.

Subsequently, we determined *Nrf2* transcription and expression to determine whether SFN plays its antioxidative role through Nrf2. The results revealed that Nrf2 expression was not obviously affected by Ang II intervention for 24 h. However, compared to the control and Ang II groups, *Nrf2* transcription and translation were significantly upregulated by SFN (Figure 3A, 3B). Considering that the phosphorylation of Nrf2 at ser40 indicates its activation, immunofluorescence staining (IF) was conducted to detect the expression and distribution of p-Nrf2. Compared to the control and Ang II groups, the high p-Nrf2 expression was observed in the nuclei of embryonic rat cardiomyocytes (H9C2) in SFN and Ang II/SFN groups (Figure 3C).

In addition, Nrf2 activation was reflected by the increased expression of antioxidant genes *NQO1*, *HO1*, and *CAT*. Therefore, their expression was detected, and found that SFN significantly upregulated the expression of *HO1*, *NQO1*, and *CAT* compared to the control and Ang II groups (Figure 3D), which was consistent with the upregulation of Nrf2 expression. These findings suggested that the protective effect of SFN on Ang II-related myocardial injury is closely related to the activation of Nrf2 rather than the inhibition of AT1 expression.

### Nrf2 gene knockdown eliminates the preventive effect of SFN on Ang II-induced myocardial damage

**Figure 4:**
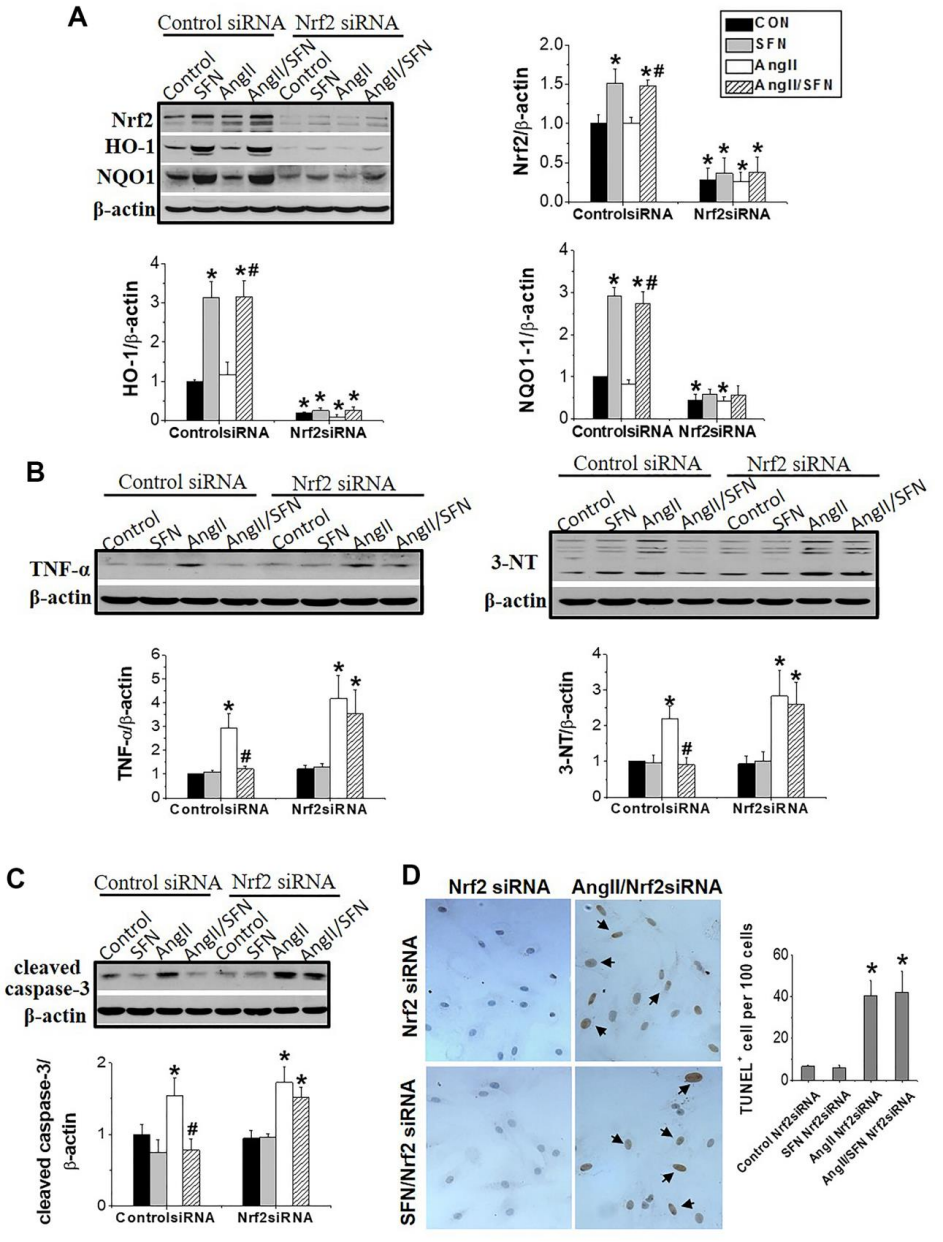
***Nrf2* gene knockdown eliminated the preventive effect of SFN on Ang II-induced myocardial damage.** Both Nrf2 knockdown cell lines and wild-type cell lines were given/not given Ang II, with/without SFN treatment. Western blot detected the expression of Nrf2 and downstream antioxidant genes NQO1 and HO-1 (**A**), TNF-α, 3-NT (**B**), cleaved caspase-3 (**C**). TUNEL staining detected apoptotic cells (**D**). Data were presented as the mean SD (n = 3). *P < 0.05 vs control; # *P* < 0.05 vs Ang II.

To explore whether Nrf2 plays a direct role in preventing SFN-mediated Ang II-induced myocardial damage, we used RNA interference technology to silence *Nrf2* in H9C2 cells. After siRNA transfection, *Nrf2* was barely expressed in the cells regardless of SFN treatment (Figure 4A). Moreover, HO-1 and NQO1 expression were significantly decreased in Nrf2 siRNA group (Figure 4A). In the Nrf2 siRNA group of H9C2 cells, Ang II enhanced the expression of the inflammatory factor TNF-α and oxidative stress indicator 3-NT, but SFN failed to downregulate the expression of these proteins (Figure 4B). Similarly, Ang II was able to induce cardiomyocyte apoptosis and the expression of the apoptotic protein caspase-3 in the Nrf2 siRNA group, but these effects were not reversed by SFN (Figure 4C, 4D).

### SFN activates myocardial *Nrf2* transcription by enhancing the histone-3 (H3) acetylation in *Nrf2* promoter region

**Figure 5:**
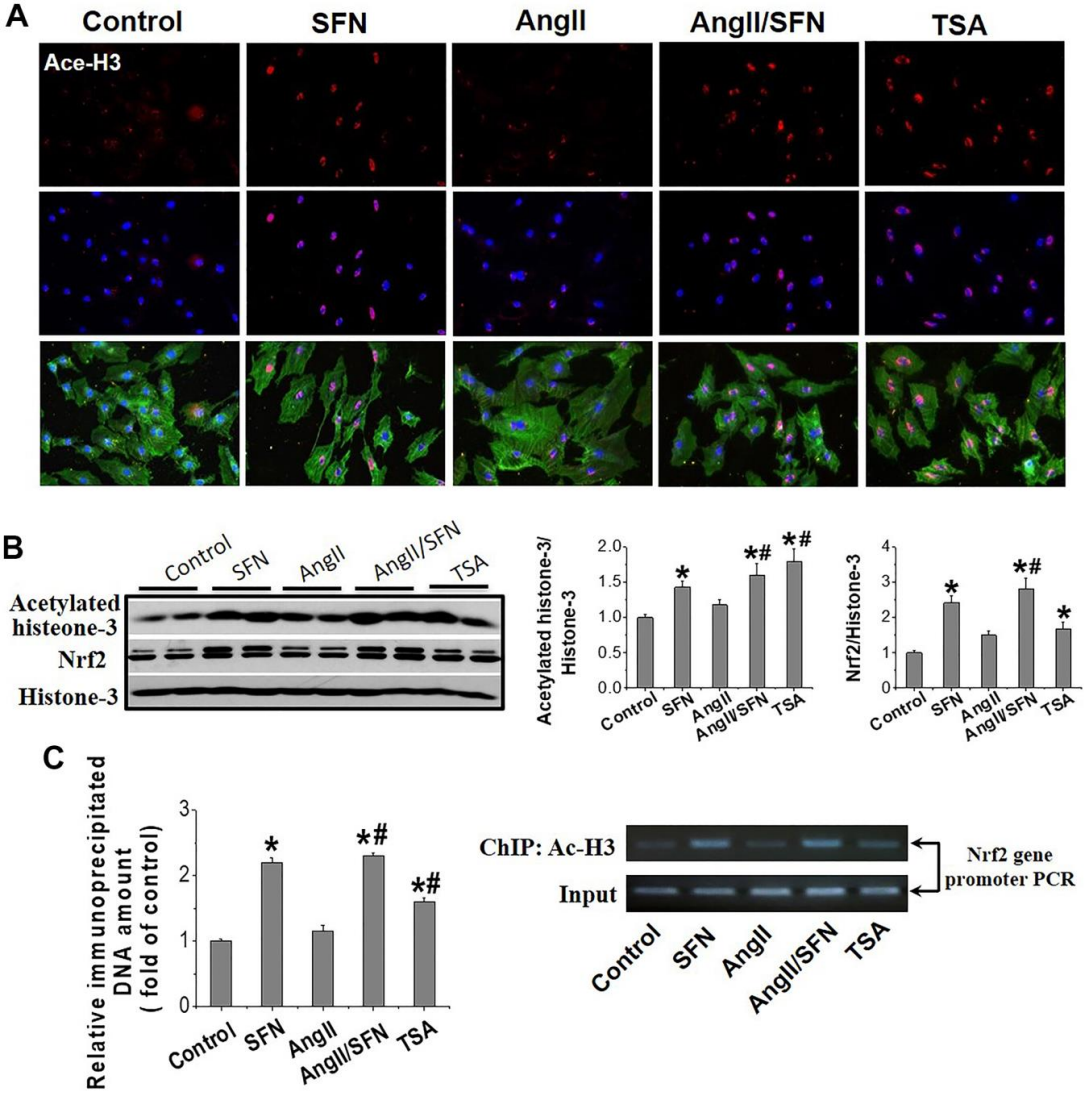
**SFN activates myocardial Nrf2 by enhancing the acetylation of histone H3 in *Nrf2* promoter region.** Immunofluorescent staining was used to detect Ace-H3 expression and distribution (red) (**A**). Western blot was used to detect Ace-H3 and Nrf2 expression (**B**). ChIP detected the enrichment of Ac- H3 in *Nrf2* promoter region. (**C**) TSA, a deacetylase inhibitor, was used as a positive control. Data were presented as the mean SD (n = 3). *P < 0.05 vs control; # *P* < 0.05 vs Ang II.

Histone acetylation loosens the chromatin structure and leads to the transcriptional activation of related genes. Trichostatin A (TSA), an HDACs inhibitor, was used as the positive control. IF showed that H3 acetylation was observed in the cardiomyocyte’s nuclei of SFN, Ang II/SFN and TSA groups (Figure 5A). Quantitative western blot analysis verified a significant increase in H3 acetylation in the SFN and Ang II/SFN groups, according to the activation of *Nrf2* (Figure 5B). Chromatin immunoprecipitation (ChIP) analysis further confirmed that SFN significantly increased H3 acetylation in *Nrf2* promoter region, which was no difference with the overall acetylation of H3 (Figure 5C). These results indicated that SFN activates Nrf2 by increasing H3 acetylation in the *Nrf2* promoter.

**Figure 6:**
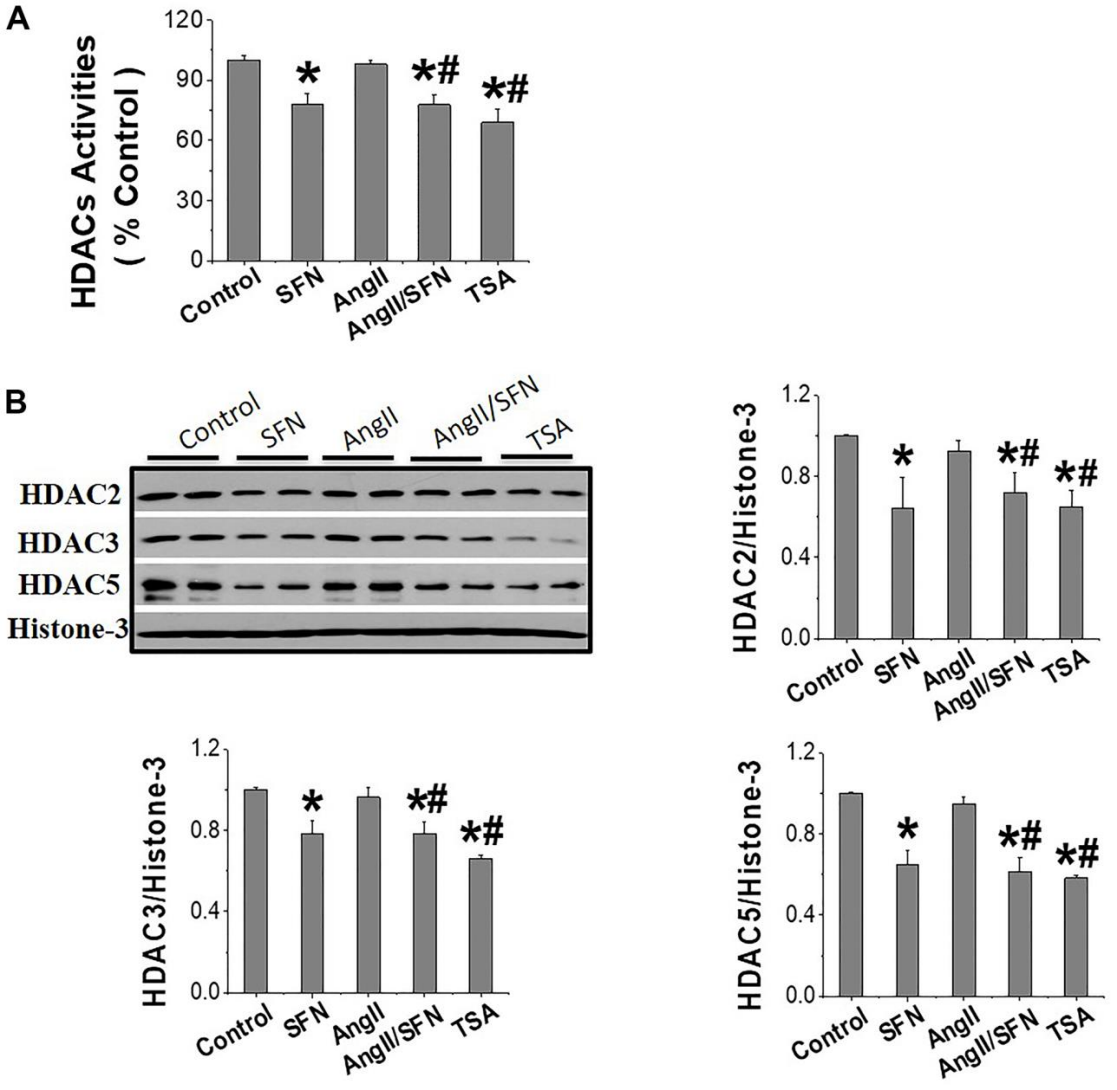
**SFN activates Nrf2 through inhibiting HDACs expression and activity.** The HDACs activity kit was used to detect the global HDACs activity (**A**). Western blot was used to detect the expression of HDAC2, HDAC3, HDAC5 (**B**). TSA, a deacetylase inhibitor, was used as a positive control. Data were presented as the mean SD (n = 3). *P < 0.05 vs control; # *P* < 0.05 vs Ang II.

HDACs jointly regulate histone acetylation levels, and it has been confirmed that SFN is an inhibitor of HDACs [35]. In view of this, our analysis revealed that SFN significantly inhibited global HDACs activity (Figure 6A) and the expression of HDAC2, HDAC3, and HDAC5 (Figure 6B) compared to the control and Ang II groups. In conclusion, these results suggested that SFN activates Nrf2 by inhibiting HDACs and increasing histone acetylation levels, which may play a crucial role in preventing Ang II-induced cardiomyocyte apoptosis.

## DISCUSSION

It has been previously confirmed that SFN protects against Ang II-induced cardiomyopathy through activating Nrf2 [25]. However, the intrinsic molecular mechanism by which SFN regulates cardiac Nrf2 and its relationship with cardiomyocyte apoptosis remain unclear. In this study, a series of *in vitro* experiments were conducted to confirm the following: (1) SFN prevents Ang II-induced cardiomyocyte apoptosis, (2) inhibition of inflammation and oxidative stress is important for the protective effect of SFN, (3) Nrf2 is a direct target of SFN’s protection on Ang II-induced cardiomyocyte apoptosis, and (4) Nrf2 activation by SFN is mediated by increased H3 acetylation through the inhibition of HDACs activity. Therefore, this study provides a theoretical basis for SFN treatment on Ang II-induced myocardial injury and provides an effective strategy for the treatment of cardiomyopathy.

Excessive activation of oxidative stress is a key cause of Ang II-induced myocardial injury [4]. In this study, we found that Ang II induced inflammation and oxidative stress, which resulted in cardiomyocyte apoptosis (Figures 1, 2). As a transcription factor for antioxidant stress, Nrf2 upregulates the expression of a series of detoxification enzymes and downstream antioxidant genes [36–38]. Moreover, downregulation of Nrf2 aggravates Ang II-induced cardiac hypertrophy [39]. In this study, we found that the upregulation of Nrf2 and the downstream antioxidant genes *NQO1*, *HO1*, and *CAT* could prevent Ang II-induced cardiomyocyte apoptosis, and the knockdown of *Nrf2* aggravates the occurrence of cardiomyocyte apoptosis (Figures 3, 4). These results suggest that Nrf2-mediated antioxidant defense mechanism plays an important role in preventing Ang II-induced cardiomyocyte apoptosis.

The synthetic Bardoxolone Methyl (BM), a Nrf2 agonist, was used in a phase III trial to prevent diabetic nephropathy, but the trial was discontinued owing to severe renal and cardiovascular toxicity in patients [40]. Subsequently, an increasing number of natural plant- and food-derived compounds, such as SFN, curcumin, and tocopherol, have shown to activate Nrf2 [41–43]. Among these, SFN has been widely studied because of its safety and non-toxicity. SFN has been shown to exert anticancer and damage-protective effects by modulating inflammation, oxidative stress, cell cycle and proliferation [44]. SFN can effectively protect testis, myocardium and aorta from diabetes-related oxidative damage through activation of Nrf2 [21, 45, 46]. Furthermore, it has been confirmed that SFN upregulates Nrf2 to prevent Ang II-induced myocardial injury *in vivo* [25]. Here, *in vitro* experiments demonstrated that SFN inhibited Ang II-induced myocardial inflammation, oxidative stress, and apoptosis (Figures 1, 2). Additionally, *Nrf2* and its downstream antioxidant genes were upregulated by SFN (Figure 3). However, SFN did not affect the expression of AT1 in the cardiomyocytes (Figure 2). Furthermore, in Nrf2 knockdown H9C2 cells, SFN did not upregulate the expression of *Nrf2* and its downstream genes and prevent Ang II-induced cardiomyocyte apoptosis (Figure 4), suggesting that SFN played a preventive role in cardiomyocyte apoptosis by directly acting on Nrf2, but not on AT1.

Previously, Nrf2 has been regulated through various ways, most notably by modifying the cysteine residues of Keap1 to induce its uncoupling [47]. Furthermore, various signalling pathways also contribute to the regulation of Nrf2, such as PI3K/AMPK, GSK-3β/Fyn and others [48, 49]. In addition, epigenetic modification is also an important means of regulating Nrf2 [50, 51]. Growing evidence suggests that SFN reactivates the transcription of the *Nrf2* gene by epigenetic modifications, including histone modification. SFN increases *Nrf2* expression by inhibiting promoter methylation of *Nrf2*, thereby significantly inhibiting TPA-induced skin carcinogenesis [34]. In a study on prostate cancer cells, SFN was revealed to increase the H3 acetylation in the *Nrf2* promoter region by inhibiting HDACs, thus enhancing *Nrf2* expression to exert the anticancer effect [33].

The HDACs family is divided into four classes. Eleven family members with highly conserved deacetylase domains are considered classic HDACs and fall into classes I, II, and IV. Class III members are known as sirtuins. Of these, HDAC1–5 are downregulated by SFN. Interestingly, SFN has tissue specificity for subtypes of HDACs that are downregulated. For example, in breast cancer cells, HDAC1–3 are inhibited by SFN to induce cell apoptosis [52, 53]; in skin cells, HDAC1–4 are regulated by SFN [34]; in the cochlea, SFN inhibits HDAC2, 4, and 5 [54]; and in colon cancer models, SFN downregulated only HDAC3 to prevent DNA damage repair [55]. Importantly, in the present study, SFN significantly inhibited HDAC2, 3, and 5 expression and HDACs activity in cardiomyocytes, thereby increasing H3 acetylation levels in the *Nrf2* promoter and upregulating *Nrf2* expression (Figures 5, 6).

**Figure 7:**
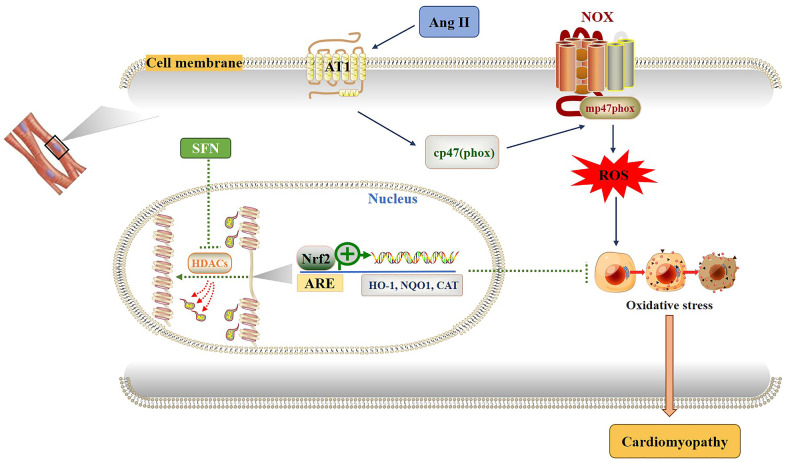
**Diagram of the mechanism by which SFN prevents Ang II-induced cardiomyocyte apoptosis.** Ang II activates oxidative stress by increasing ROS leading to inflammation, oxidative stress and fibrosis in cardiomyocytes. SFN prevent Ang II-induced cardiomyocyte apoptosis by inhibiting HDACs to activate Nrf2 and downstream antioxidant genes.

In conclusion, SFN plays a protective effect in Ang II-induced cardiomyocyte apoptosis by inhibiting HDACs and increasing the expression of *Nrf2* and downstream genes (Figure 7). Therefore, SFN or other Nrf2 activators may be promising candidates for treating oxidative stress-related diseases. Recently, clinical trials have shown that SFN-rich broccoli sprouts are safe and effective in treating allergic asthma and sickle cell disease [56, 57]. However, other clinical trials have revealed that the Nrf2 activator BM shows cardiotoxicity when treating diabetes mellitus [58], and resveratrol shows no effect on chronic kidney disease [59]. It is still believed that drug development of Nrf2 activators, such as SFN, and clinical trials are worthy of further investigation.

## MATERIALS AND METHODS

### Cell culture

H9C2 cells were purchased from ATCC (CRL-1446, MD, Manassas, VA, USA) as previously described [25] and cultured in Dulbecco’s modified Eagle medium. The ambient CO_2_ concentration was 5% and the temperature was 37° C. SFN (100 nmol/L, Sigma-Aldrich) was added and incubated for 24 h [21].

### RNA interference experiment

H9C2 cells were inoculated in 6-well plates and replaced with antibiotic-free medium one day before transfection. Transfection was carried out when the cells reached 70–90% confluence. According to the instructions of Lipofectamine™ 2000 (Invitrogen) reagent, 50 μL OPTI-MEM® was applied to dilute 1.0 μg DNA and 1.5 μL reagent. The two solutions were mixed, kept at room temperature for 10 min, then added to the wells, and mixed well. The culture medium was replaced 6 h after transfection, and *Nrf2* expression was detected after two days of continued culture.

### qPCR

qPCR was used to analyze the expression of *Nrf2*, *AT1*. After washing the medium with PBS, 1 mL TRIzol was added, and kept at room temperature for 10 min. Total RNA was extracted using the TRIzol reagent and used to synthesize cDNA using the TransGen Biotech kit. qPCR analysis was performed using the Applied Biosystems PRISM 7700 Quantitative PCR instrument, Brilliant II SYBRs Green PCR Master Mix (Agilent Technologies), and specific primers (Nrf2: Mm00477784; AT1: Mm00616371; Applied Biosystems). β-actin was used as an internal control.

### Western blot analysis

Cells were lysed with radio-immunoprecipitation assay (RIPA) lysate containing phenylmethanesulfonyl fluoride (PMSF) at a volume ratio of 1:10 (sample: lysate) and homogenized on ice. Then, the cell lysate was centrifuged at 12000 × *g* for 30 min. The protein concentration was measured by the bicinchoninic acid assay (BCA) method. SDS-PAGE was performed and proteins were transferred to PVDF membranes, which were then blocked with 5% skim milk for 1 h. Membranes were incubated at 4° C overnight with primary antibodies, including cleaved caspase-8, cleaved caspase-3, 4-HNE, 3-NT, TNF-α, NF-κB, IKβ, p-P47^phox^, AT1, Nrf2, p-Ser40-Nrf2, HO-1, NQO1, CAT, and HDACs subtypes. The membranes were then incubated with horseradish peroxidase-labeled secondary antibodies (1:2000) diluted with TBST for 1 h at room temperature. Color development exposure (ECL ultrasensitive color development solution) and protein expression were observed. The Image J software (v1.8.0) was used to analyze the quantitative densitometry of the bands.

### TUNEL staining

TUNEL staining was performed using the Kit (Millipore) according to the instructions. The cell slides were digested with 20 μg/mL protease K and blocked with 50 μL H_2_O_2_ at room temperature for 15 min. After washing, equilibration buffer was added dropwise for 5 min, 27 μL of working solution (TDT enzyme) was added and incubated for 1 h at 37° C in the dark, and the reaction was terminated by adding 50 μL reaction termination solution for 5 min at room temperature. Then anti-digoxigenin conjugate was added and incubated at room temperature for 30 min. DAB was developed for color, nuclei were stained using Mayer’s hematoxylin, gradient alcohol was used for dehydration, xylene was used for transparency, and the slides were sealed and observed under a microscope. Ten fields were selected per section. Cells with brownish yellow particles in the nuclei were considered apoptotic cells. The number of positive cells and total number of cells in a field were counted using a microscope (400×) to calculate the apoptosis rate.

### IF

The cells were fixed with 4% formaldehyde and then underwent cell permeation. Serum was used to block non-specific binding, and the primary antibodies (p-ser40-Nrf2, Ace-H3) were added. The cells were washed, titrated with fluorescently labeled secondary antibody, blocked with glycerol, and microphotographed for analysis.

### ChIP assay

The assay was performed using the EpiquikTM Chromatin Immunoprecipitation Kit (Epigentek) according to the instructions. H9C2 cells were incubated with 1% formaldehyde for 10 min, glycine was used to terminate cross-linking, and then 400 μL SDS lysis solution containing a protease inhibitor. Cells underwent ultrasound crushing and centrifugation to collect the supernatant. ChIP dilution buffer, 20 μL 50× IPC, and 60 μL Protein A Agarose/salmon sperm DNA mixture were added to 100 μL of the supernatant, mixed at 4° C for 3 h, and centrifuged to obtain the supernatant. Monoclonal antibodies (2 μL) against acetylated H3 were added to the supernatant and incubated overnight at 4° C. The precipitate was collected by centrifugation and washed with solutions in the following order: TSI, TSII, Buffer III, and TE solutions. The eluent was then added and the supernatant was collected by centrifugation. NaCl (5 mol/L) was used to unlock the crosslinking, and the DNA was extracted and recovered. PCR was performed using *Nrf2* promoter region-specific primers: 5'-AGGGTCACAGCATTAGG-3' (sense); 5'-ACAGGGTTCCTTTCCAT-3' (antisense).

### HDACs activity assay

The nuclei of H9C2 cells were extracted using the kit (Nanjing KGI Biotechnology Co., Ltd.), and SDS lysis solution containing protease inhibitors was added to extract the nuclear proteins. The EpigenaseTM HDACs colorimetric activity/inhibition direct assay kit (Epigentek) was used according to the instructions. Briefly, 40 μg water was added to 50 μg nuclear protein sample and incubated with 10 μL HDACs substrate and 10 μL buffer solution at 37° C for 90 min. After washing the plates, the detection antibody HO-5 and chromogenic solution were added to each sample, and the OD value at 450 nm was determined. According to the formula, HDACs activity (OD/min/mg) was calculated and compared with the control group to determine the relative activity of HDACs.

### Statistical analysis

Two-way ANOVA was performed to compare differences among multiple groups, and the Tukey’s test was performed to compare differences between two groups. *P* < 0.05 was considered statistically significant.
